# Les calculs urinaires de l'enfant au Burkina Faso: à propos de 67 cas

**DOI:** 10.11604/pamj.2015.20.352.4407

**Published:** 2015-04-14

**Authors:** Isso Ouédraogo, Aïcha Madina Napon, Emile Bandré, Francis Somkieta Ouédraogo, Wendlamita Toussaint Tapsoba, Albert Wandaogo

**Affiliations:** 1 Service de Chirurgie Centre Hospitalier Universitaire Pédiatrique Charles de Gaulle Ouagadougou Burkina Faso; 2 Service de Radiologie et de Radiodiagnostic Centre Hospitalier Universitaire Pédiatrique Charles de Gaulle Ouagadougou Burkina Faso

**Keywords:** Lithiases urinaires, enfant, symptômes, diagnostic, composition chimique, traitement, Burkina Faso, urolithiasis, child, symptoms, diagnosis, chemical composition, treatment, Burkina Faso

## Abstract

L'objectif de cette étude est de déterminer la fréquence, de décrire les circonstances de découverte, les signes cliniques et paracliniques, la composition chimique des calculs prélevés et les difficultés rencontrées dans le traitement des lithiases urinaires. Notre étude a été rétrospective sur une période de six ans (janvier 2005 à décembre 2010) et a eu pour cadre le CHUP-CDG et a concerné 67 patients âgés de moins de 15 ans opérés pour lithiases urinaires. Les calculs de la dernière année au nombre de douze ont fait l'objet d'une analyse spectrophotométrique. La lithiase urinaire figure parmi les dix premières pathologies du service de chirurgie et représente 1,32% des hospitalisations. L’âge moyen de nos patients est de deux ans et varie de 6 mois à 14 ans. La symptomatologie de la lithiase urinaire est polymorphe. Le diagnostic des lithiases urinaires a été essentiellement radiologique (ASP) dans 87, 50 des cas. Les localisations les plus fréquentes sont: vésicales (49,25%) et pyéliques (46,26%). L'ECBU a révélé une infection urinaire chez 9 patients. Les germes le plus fréquemment rencontrés sont: Klebsiella pneumoniae pneumoniae (22,22%) et staphyloccocus aureus (22,22%). Les difficultés du traitement sont dues à la modicité de nos moyens diagnostiques et à la nature chimique des calculs et le traitement a été dans tous les cas chirurgical. La composition chimique est dominée par les sels calciques notamment les oxalates, les phosphates et les carbonates.

## Introduction

Le terme lithiase urinaire ou calcul urinaire anciennement appelé « maladie de la pierre » désigne la maladie qui est caractérisée par la formation de concrétions (calculs) dans les reins ou dans les voies excrétrices urinaires [1]. Au sein des populations dont le niveau socio-économique est faible, la lithiase touche essentiellement les enfants, avec un rapport garçons/filles très élevé. La localisation anatomique initiale des calculs, qui est plutôt vésicale au sein des populations à faible revenu est essentiellement rénale dans les populations de niveau socio-économique moyen ou élevé. La nature de ces calculs est phosphatique ou urique (et uratique) dans les populations à faible revenu, et calcique dans les pays industrialisés. La lithiase urinaire de l'enfant est caractérisée par un taux de récidive plus élevé que chez l'adulte (10 à 48% vs 7%) [1, 2]. Qu'en est-il chez nous? L'objectif de cette étude est de déterminer la fréquence, de décrire les circonstances de découverte, les signes cliniques et paracliniques, de déterminer la composition chimique des calculs prélevés et de décrire les difficultés rencontrées dans le traitement des lithiases urinaires.

## Méthodes

Cette étude est rétrospective a été réalisée de janvier 2005 à décembre 2010; elle a eu pour cadre le service de chirurgie pédiatrique du CHUP-CDG. Elle a intéressé tous les enfants de moins de 15 ans admis au CHUP-CDG par les urgences chirurgicales ou le service des consultations externes pour lithiases urinaires. Pour chaque patient, une fiche a été remplie et portant les différentes informations concernant le sexe, l’âge, la profession des parents, la zone de résidence, les circonstances de découverte, les antécédents personnels, les antécédents familiaux, la localisation anatomique, le nombre de lithiases et le mode d’élimination du calcul. Les explorations comprenaient la biologie, la radiologie des voies urinaires associant une échographie et une urographie intraveineuse, de même qu'un examen cytobactériologique des urines ont été réalisés lors de l'admission chez certains patients. Les calculs recueillis durant la dernière année de l’étude (de janvier 2010 à décembre 2010) ont été analysés par spectrophotométrie d'Absorption Atomique (AAS). Elle comportait dans un premier temps un examen sous une loupe pour déterminer le type morphologique du calcul et dans un deuxième temps, les calculs ont été analysés après dissolution par de l'acide pour déterminer la composition en ions, à défaut de la spectrophotométrie à infrarouge.

## Résultats

### Caractéristiques sociodémographiques

**Figure 1:**
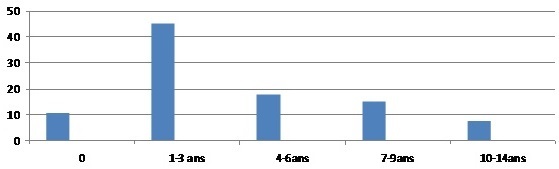
Répartition des malades selon les tranches d’âge

Nous avons recruté 67 enfants porteurs de lithiases urinaires, soit 1,32% des 5066 patients admis dans le service de chirurgie pendant la même période. Notre échantillon était composé de 60 garçons (89,55%) et 7 filles (10,45%); soit un sex-ratio de 8,57. L’âge des patients variait de 0 à 14 ans avec une moyenne de 2 ans. Les enfants lithiasiques de trois ans et plus représentaient plus de la moitié des patients soit 70,14% (Figure 1).

### Données cliniques

Le mode d'accès des patients dans le service a été dominé par les entrées en urgence (64,29%) soit plus de la moitié des patients. Les principaux motifs de consultation ont été: rétention aigue d'urine (29,85%) soit 20 cas; colique néphrétique (17,91%) soit 12 cas; douleurs abdominales chroniques (4,47%) soit 3 cas; anurie (4,47%) soit 3 cas; hématurie (2,98%) soit 2 cas; pyurie (1,49%) soit 1 cas.

### Evolution

L’évolution de la lithiase chez nos patients a été émaillée de complications dans 38,80% des cas: hydronéphrose dans 12 cas, urétéro-hydronéphrose sur 2 cas de calculs enclavés, 2 cas d'anurie, 3 cas d'infections basses, deux pyélonéphrites, une pyonéphrose, un abcès périnéphritique.

### Données para cliniques

**Figure 2:**
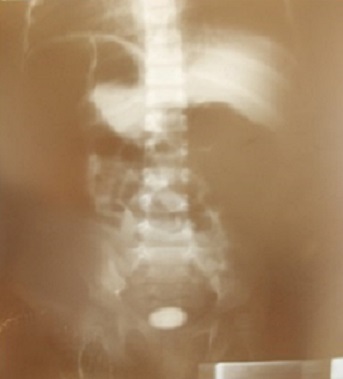
Lithiase vésicale stratifiée

Dans 6 cas, soit 8,96%, l'urée et la créatinine sanguine étaient élevées; il s'agissait de lithiases pyéliques obstructives engendrant une souffrance rénale. L’électrophorèse de l'hémoglobine n'a été demandée que chez 6 patients et n'a révélé que 2 cas de trait drépanocytaire AC. Le Potentiel d'Hydrogène (PH) urinaire a été demandé chez 2 malades. Ces résultats étaient respectivement 6 et 6,5. L'examen cytobactériologique des urines a été demandé chez 15 patients et les résultats ont été positifs dans 60% des cas (9 malades) (Tableau 1). *Klebsiella pneumoniae et Staphyloccocus aureus* sont les germes les plus rencontrés. La cristallurie a mis en évidence des oxalates de calcium, de nombreux cristaux de triphosphates, carbonates de calcium, acide urique (Tableau 2). L'imagerie diagnostique a été réalisée 109 fois; il s'agissait de la radiographie de l'abdomen sans préparation (ASP) 56 cas, l’échographie abdominale 50 cas et de l'urographie intraveineuse (UIV) 3 cas. L'ASP a confirmé le diagnostic de lithiase urinaire dans 87,5% soit 49 cas (Figure 2). L’échographie a confirmé le diagnostic de lithiase urinaire dans 100% des cas soit 50 cas. L'UIV a contribué au diagnostic dans 3 cas d'anurie calculeuse. La localisation vésicale prédominait, 33 cas soit 49,25%; suivie de la localisation pyélique avec 31 cas soit 46,26% et s'observaient chez les enfants âgés de 1 à 3 ans; 58,20% des calculs extraits étaient uniques contre 10,44% où le nombre de calculs était supérieur à trois (Tableau 3). La consistance des calculs n’était pas précisée dans 80,59%. Cependant dans 17,91% des cas, ils étaient durs. Il en est de même pour la surface où les calculs rugueux prédominaient. Les dimensions étaient variées; la plus grande était supérieure à 3 cm. Le poids des calculs était divers, certains calculs était très volumineux: les plus gros sont des calculs vésicaux et un pesait jusqu’à 5,74g (Tableau 4).

**Tableau 1:** Les germes isolés

Germes	Effectif	%
*Klebsiella pneumoniae*	2	22,22
*Staphyloccocus aureus*	2	22,22
*Eschérichia coli*	1	11,11
*Pseudomonas spp*	1	11,11
*Enterobacter sp*	1	11,11
*Salmonelle de groupe B*	1	11,11
*Candidas albicans*	1	11,11
**Total**	**9**	**100**

**Tableau 2:** Les cristaux isolés

Cristaux	Effectif	%
Oxalate de Ca	2	40
Triphosphate	1	20
Carbonate de Ca	1	20
Acide urique	1	Rares
**Total**	**5**	**100**

**Tableau 3:** Localisation selon l’âge

Age (an)	Pyélique	Urétérale	Vésicale	Urètre	Total
<1	4	0	2	0	6
1-3	13	3	15	3	34
4-6	2	0	8	1	11
7-9	4	0	5	1	10
10-14	8	1	3	0	12
Total	31	4	33	5	73

**Tableau 4:** Conversion des scores de la spectrophotométrie

	L1	L2	L3	L4	L5	L6	L7	L8	L9	L10	L11	L12	%
**Lithiasis**													
Calcium	83,4	72,3	91,7	87,1	67,9	81,2	86,4	81,3	81,8	51,5	59,5	91,1	77,9
Magnesium	8,4	8,1	1,8	2,1	4,9	5,8	3,3	4	3,5	3,7	3,1	0,5	4,1
Ammoniac	3,1	5,8	2,2	2,3	27,1	10,5	6	5,3	6	6,3	6	2	6,8
Phosphates	5,1	13,8	4,3	7,6	0,1	2,5	4,3	9,4	8,7	38,5	31,4	6,4	11,2

NB: La technique n’était pas adaptée à l'analyse des oxalates et carbonates

### Traitement

**Figure 3:**
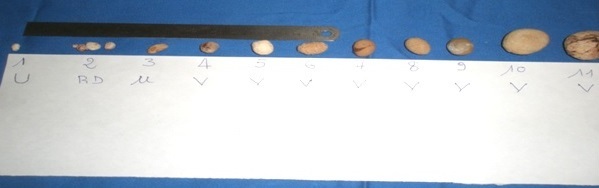
Calculs recueillis au bloc opératoire du CHUP-CDG

La chirurgie a été notre seule voie de recours pour extraire les calculs des voies urinaires et cela s'est faite sous anesthésie générale (Tableau 5). La cystotomie et la pyélotomie ont été les principales voies d'abord mais il faut noter que dans les cas où les calculs étaient enclavés au niveau de l'urètre pénien nous avons eu recours à l'extraction par les instruments (Figure 3).

**Tableau 5:** Modes d'extraction

Modes d'extraction		Effectif	%
ombotomie	Pyélotomie	31	53,35
	Urétérostomie	4	7,14
Cystotomie		33	58,93
Urétrotomie		2	1,78
Instrumental		3	5,35
Total		67	100

### Evolution

Les suites opératoires ont été simples dans 95,53%; cependant nous avons noté quelques complications: urétérostomie bilatérale dans 1 cas, un cas de fistule scrotale, un cas de néphrectomie.

## Discussion

La lithiase urinaire chez l'enfant comme chez l'adulte présente une grande variabilité de fréquence suivant les pays: notre étude a rapporté 67 cas de lithiases opérées dans le service de chirurgie en 5 ans soit 1,32% des activités chirurgicales; au Mali Ouattara [3] en 4 ans a observé 146 cas tout âge confondu; en Tunisie 205 cas pédiatriques ont été colligés en 14 mois [4] contre 420 cas pédiatriques en 6 ans en France [1]. Notre échantillon était composé de 60 garçons (89,55%) et 7 filles (10,45%); soit un sex-ratio de 8,57. La classe d’âge la plus touchée était les enfants de 0 à 3 ans qui représentaient plus de la moitié des malades admis dans le service soit 59,7%. Le plus jeune avait 6 mois. En Tunisie, le plus jeune avait 5 mois [2]. Notre étude a montré une large prédominance du sexe masculin avec 60 cas soit 89,55% sur le sexe féminin 7 cas soit 10,45%. Nos résultats sont conformes à ceux retrouvés dans la littérature [1, 2, 5–9]. Cette prédominance peut s'expliquer par la longueur de l'urètre (facteur de rétention) chez le garçon alors que la brièveté de l'urètre féminin et la puissance du jet urinaire permettent à la fille d’éliminer plus facilement ses calculs. Nos résultats démontrent le polymorphisme des signes et surtout présentent une particularité: la rétention est le symptôme le plus fréquent 20 cas soit 29,85% alors que dans la littérature ce sont la colique néphrétique et l'hématurie qui prédominent [2, 5, 10–12]. Nous expliquons ce phénomène par la longueur de l'urètre (facteur de rétention) et la fréquence plus élevée des calculs vésicaux chez le garçon. Mais aussi nous sommes dans un contexte socioculturel particulier où les parents vont plutôt s'inquiéter pour la rétention aiguë d'urine que pour les coliques néphrétiques qui avec leur aspect chronique et intermittent font de ces petits patients des enfants plaintifs; ceci finit par agacer l'entourage qui prête moins d'attention aux complaintes de l'enfant. Les parents vont alors être plus prompts à conduire un enfant présentant une rétention aiguë d'urine à l'hôpital. Egalement le contexte de pauvreté fait que ces parents ne vont se présenter à l'hôpital que si l'urgence les y oblige. Dans notre série, la lithiase a engendré, 12 cas d'hydronéphrose, la pyurie a été rencontrée dans 1 cas, l'anurie calculeuse 2 cas, la pyélonéphrite retrouvée par 2 fois, 1 cas de pyonéphrose, 1 cas d'insuffisance rénale aiguë. Ce résultat s'explique par le délai tardif de consultation dans notre série. Aucun bilan phosphocalcique n'a été réalisé dans notre série afin qu'on puisse évoquer une cause métabolique pouvant expliquer la lithogénèse. Nous n'avons donc pas mis en évidence une cause générale responsable du trouble métabolique chez nos patients.

L'uroculure réalisée chez 15 patients dans notre étude a révélé une infection urinaire dans 9 cas soit 60%. Les germes les plus fréquemment rencontrés ont été: le klebsiella et le staphyloccocus. Les lithiases infectieuses ont représenté 9 cas. La relation entre infection urinaire et lithiase a été établie et retrouvée dans plusieurs études [4, 11]. Alaya et al dans une série de 168 patients ont noté 24 urocultures positives et dans la moitié des cas, c'est *protéus mirabilis* qui a été isolé [13]. L'ASP fait chez 56 patients a mis en évidence la lithiase urinaire chez 49 patients soit 87,50%. Cet examen est le plus accessible dans nos conditions de pratique en urgence tant du point de vue de son coût que de sa disponibilité. Dans toutes les séries, le diagnostic de la majorité des lithiases urinaires a été fait par l'ASP [4, 6, 12, 14]. Ceci s'explique par la radio-opacité de la majorité des lithiases urinaires. Ce ne sont pas tous les calculs qui sont radio-opaques mais cet examen nous est d'un secours appréciable dans les rétentions aiguës d'urine reçues en urgence. L'Echographie faite chez 50 patients a révélé la présence de la lithiase chez tous les patients, elle a l'avantage de poser le diagnostic mais aussi de renseigner sur l’état du parenchyme rénal qui est souvent mis à mal par la présence du calcul. Elle a révélé un cas d'abcès périnéphritique, une pyonéphrose, 12 cas d'hydronéphrose. Des uropathies malformatives telles les duplications pyélo-urétérales, des urétérocèles n'ont pas été mises en évidence. La confirmation de la lithiase urinaire a été faite par l'UIV chez 3 patients soit 4,47%. L'UrétroCystographieRétrograde et la cystoscopie n'ont pas été faites chez nos patients du fait de l'indisponibilité de ces méthodes exploratrices.

Dans notre série la localisation des lithiases urinaires étaient vésicales dans 49, 25% puis pyéliques dans 46,26%. Oussama et al. ont noté que la moitié des calculs étaient localisés dans la vessie (51,1%) [15]. La prédominance de la localisation basse de ces lithiases situe notre population d’étude parmi les moins nanties. Alaya et al en Tunisie ont trouvé une distribution proche de celle des pays développés marquée par une prédominance des localisations rénales (51,2%) contre seulement 30,9% pour celles à localisation vésicale [5]. La localisation multiple des lithiases a concerné 9 malades (6 cas de lithiases pyéliques bilatérales, 1 cas de lithiase pyélique gauche multiple, 1 cas de lithiase pyélique bilatérale multiple et 1cas de lithiase pyélique bilatérale associée à une lithiase vésicale) soit 13,43% de l'effectif chez qui nous suspectons une lithiase d'organisme qui aurait pu être confirmée par le bilan métabolique. Il ressort de l'analyse de la composition chimique des calculs que les sels les plus fréquemment rencontrés, par ordre d'importance numérique croissante sont les phosphates, les oxalates, les carbonates et les urates. Dans la majorité des cas il s'agit de sels de calcium (oxalate de calcium, phosphate de calcium et carbonate de calcium) qui ont représenté 74,17% des Ions suivis des phosphates ammoniaccomagnésiens (4,11%). Dans notre série seulement 12 lithiases ont bénéficié d'une analyse spectrophotométrique et cet échantillon réduit ne nous permet pas de faire des comparaisons. Une étude multicentrique réalisée par Laziri et al. au Maroc a montré que la composition de ces calculs était différente suivant les régions géographiques [16]. Les ECBU (cristallurie) ont mis en évidence la présence dans les urines de certains de nos patients: l'oxalate de calcium (2 cas), de nombreux cristaux de triphosphates de calcium (1 cas), des carbonates de calcium (1 cas) et l'acide urique (rare) 1 cas; ce qui témoigne bien de leur existence. Nous n'avons pas pu les mettre en évidence par la technique utilisée qui emploie des acides forts qui détruisent certaines substances organiques (Acide sulfurique et acide fluorhydrique). La présence de l'ammoniac (6,9%) dans la composition des calculs analysés supposent l'existence de phosphates ammoniaccomagnésiens (P.A. M). Nous n'avons pas pu rechercher la cystine qui demande une technique plus performante.

Le traitement chirurgical a été le seul moyen dont nous avons disposé pour extraire les lithiases. Les méthodes non invasives telles que la cystoscopie et la lithotripsie extracorporelle sont absentes de notre arsenal thérapeutique. Même si nous en disposions les indications seraient restreintes; nos patients sont vus tard avec des lithiases de la taille de 3 cm et pesant 5,74 g. La plupart de ces patients sont reçus dans le cadre de l'urgence notamment les rétentions aiguës d'urine et les coliques néphrétiques et leur prise en charge ne permet pas d'explorations plus étendues qui pourraient orienter mieux le choix thérapeutique. Cette situation ne laisse pas de place à un traitement médical et notre recours ne peut être que chirurgical. Il a été le seul moyen de traitement curatif dans notre service. Tous les patients ont subi une ablation chirurgicale de leur calcul urinaire. Jellouli et al. en Tunisie ont également eu recours à la chirurgie pour 60 malades et seulement 2 autres ont été traités par voie endoscopique associée à une lithotritie balistique endocorporelle [5]. Comme traitement médical à but préventif, en dehors des antalgiques, anti-inflammatoires et antispasmodiques aucun de nos patient n'a été mis sous alcalinisant (Bicarbonate de sodium) ni acidifiant (Acide ascorbique) puisque le pH urinaire n'est pas demandé systématiquement (2 cas dans notre série sur 67 patients). Enfin nous signalons que peu de nos patients ont fait le bilan phosphocalcique, l'ECBU et le pH urinaire à cause de leurs moyens financiers très limités.

## Conclusion

La lithiase urinaire est une pathologie qui représente 1,32% des hospitalisations. Les principaux facteurs favorisants de la lithiase urinaire de l'enfant sont: les infections urinaires, l'alimentation, le climat chaud avec l'insuffisance de l'apport hydrique. L'hydronéphrose a été la plus fréquemment rencontrée 12 cas soit 17,91%. La chirurgie à ciel ouvert a été pratiquée dans tous les cas parce que les lithiases étaient soient mal tolérées (colique néphrétique) ou provoquant une rétention soient dues à une complication (anurie calculeuse, insuffisance rénale…). Nous n'avons pas pu instituer un traitement étiologique parce que le bilan phosphocalcique n'a pas été réalisé et aucun patient n'a pu faire l'analyse chimique de la lithiase. L'analyse spectrale des calculs nous a donné le profil de leur composition qui ne diffère pas de loin de celle rencontrée dans certains pays.
